# Decellularized small intestine submucosa/polylactic-co-glycolic acid composite scaffold for potential application in hypopharyngeal and cervical esophageal tissue repair

**DOI:** 10.1093/rb/rbaa061

**Published:** 2021-03-13

**Authors:** Shijie Qiu, Lijin Liang, Peng Zou, Qi Chen

**Affiliations:** 1 Department of Otorhinolaryngology-Head and Neck Surgery, The First Affiliated Hospital of Zhejiang University, 79 Qingchun Road, Hangzhou, 310003, People’s Republic of China; 2 Department of Otorhinolaryngology-Head and Neck Surgery, Lihuili Hospital of Ningbo University, 57 Xingning Road, Ningbo, 315041, People’s Republic of China; 3 Ningbo Regen Biotech Co., Ltd, 199 East Hexiao Road, Ningbo, 315157, People’s Republic of China

**Keywords:** small intestine submucosa, polylactic-co-glycolic acid, hypopharyngeal and cervical esophageal cancer, tissue repair, composite scaffold

## Abstract

There has been an increase in the incidence of hypopharyngeal and cervical esophageal cancer worldwide, and hence growing needs for hypopharyngeal and cervical esophageal tissue repair. This work produced a bi-layer composite scaffold with decellularized small intestine submucosa and polylactic-co-glycolic acid, which resembled the layered architectures of its intended tissues. The decellularized small intestine submucosa contained minimal residual DNA (52.5 ± 1.2 ng/mg) and the composite scaffold exhibited satisfactory mechanical properties (a tensile modulus of 21.1 ± 4.8 MPa, an ultimate tensile strength of 14.0 ± 2.9 MPa and a failure strain of 26.9 ± 5.1%). The interactions between cells and the respective layers of the scaffold were characterized by CCK-8 assays, immunostaining and Western blotting. Desirable cell proliferation and phenotypic behaviors were observed. These results have provided an important basis for the next-step *in vivo* studies of the scaffold, and bode well for its future clinical applications.

## Introduction

Due to the environmental issues, the excessive consumption of alcohol and tobacco, and in some regions the habit of chewing areca nuts, there has been an increase in the incidence of hypopharyngeal and cervical esophageal cancer worldwide [1]. A considerable percentage of the patients with those tumors require laryngopharyngectomy and/or esophagectomy, which would inevitably cause tissue loss. Large defects are commonly repaired by autologous myocutaneous flaps and intestinal segments, respectively [2]. These procedures are relatively destructive and may cause secondary conditions to patients, a problem for which the surgeons and researchers have indeed been seeking for suitable materials to address.

Both the hypopharyngeal and cervical esophageal tissues exhibit layered architectures. The hypopharyngeal wall consists of a mucosal layer, a fibrous layer, a muscular layer and a layer of fascia, while the cervical esophageal wall is comprised of a mucosal layer, a submucosal layer, a muscular layer and an external fibrous layer [3]. It is logical to respect these layered structures when developing materials for repairing these tissues, and various layers have already been mimicked. Shen *et al.* [4] seeded epithelial cells and fibroblasts on poly(ester urethane) scaffolds to replicate the mucosal and fibrous layers of hypopharynx. Kuppan *et al.* [5] co-cultured epithelial cells and smooth muscle cells on poly(3–hydroxybutyrate–co–3–hydroxyvalerate) (PHBV) and poly(ɛ-caprolactone) (PCL) scaffolds, and achieved distinctive mucosal and muscular layers for esophagus. Hou *et al.* [6] developed a bi-layer scaffold for esophageal tissue with crosslinked polyurethane as the muscular layer, and acellular esophageal mucosa as the mucosal layer.

The authors’ attention was particularly drawn to the reported findings that small intestine submucosa (SIS) promoted smooth muscle regeneration in organs such as stomach and bladder [7–9], and that polylactic-co-glycolic acid (PLGA) supported proliferation and organization of epithelial cells, as well as formation of glands [10, 11]. Consequently, a bi-layer composite scaffold was proposed by the authors for hypopharyngeal and cervical esophageal tissue repair, with decellularized SIS intended for the muscular layer and PLGA nanofibers intended for the mucosal layer.

This study therefore assesses the physical and biochemical properties of the composite scaffold, serving as a forwarding step toward future *in vivo* evaluation of its intended clinical applications.

## Materials and methods

### Reagents

Sodium dodecyl sulfate (SDS), Triton X-100 and trypsin were purchased from Sangon Biotech (Shanghai) Co., Ltd. PLGA (LA:GA = 85:15, ŋ = 3.1DL/g) was purchased from Corbion Trading (Shanghai) Co., Ltd. 2,2,2-trifluoroethanol (purity 99.5%) was purchased from Shanghai Aladdin Bio-Chem Technology Co., Ltd. GenElute™ Mammalian Genomic DNA Miniprep Kit and radio immunoprecipitation assay (RIPA) buffer were purchased from Sigma-Aldrich. Quant-iT PicoGreen dsDNA Assay Kit and 4′,6-Diamidine-2-phenylindole (DAPI) were purchased from Invitrogen. Epithelial cell medium and smooth muscle cell medium were purchased from Sciencell Research Laboratories, Inc. Cell Counting Kit-8 (CCK-8) was purchased from Dojindo. Hematoxylin and Eosin Staining Kit (H&E) and bicinchoninic acid (BCA) protein assay kit were purchased from Shanghai Beyotime Biotechnology Co., Ltd. Anti-Cytokeratin-14 (CK-14) antibody, anti-actin antibody and horseradish peroxidase (HRP)-conjugated secondary antibody were purchased from ABCam.

### Preparation of decellularized small intestine submucosa (dSIS)

The small intestines were harvested from healthy pigs (each weighing approximately 100 kg at 6 months) within 4 h after slaughter. The serosa, muscularis and mucosa were mechanically removed, and the remaining submucosa was rinsed with deionized water. Decellularization process was performed according to published protocols [12, 13]. The SIS was sequentially treated with: (i) acetone and ethanol (2:1, v/v) for 4 h, (ii) 0.05% trypsin and 0.02% EDTA at 37°C for 8 h, (iii) 4% SDS and 3% Triton X-100 for 24 h and (iv) 0.1% peroxyacetic acid and 20% ethanol for 30 min. Finally, the dSIS was thoroughly rinsed with deionized water to remove residual reagents, and lyophilized.

### Histological analysis

Histological analysis was performed to evaluate the efficiency of the decellularization procedure. The dSIS was fixed in 4% paraformaldehyde followed by paraffin embedding. The specimens were cut to 5 μm thickness and stained with H&E for observation.

### DNA quantification

DNA was extracted and purified using GenElute™ DNA Miniprep Kit following the manufacturer’s recommended protocol. Briefly, the SIS and dSIS samples were completely digested with Proteinase K at 55°C, followed by cell lysis at 70°C for 10 min. Ethanol (95–100%) was added to the lysate, and the lysate was then transferred into the binding column. After two washes with the wash solution, the DNA was eluted by Tris–EDTA buffer from the binding column. The concentration of each extracted DNA sample was determined using Quant-iT PicoGreen dsDNA Assay Kit. Manufacturer’s recommended protocol was also followed. Briefly, a standard curve was firstly established by preparing samples of known DNA concentrations from 0 to 1000 ng/mL. DNA samples from SIS and dSIS were diluted so that their absorbencies would fall into the linear region of the standard curve. Quant-iT PicoGreen reagent was then added to the samples and allowed to react for 5 min. Fluorescence intensity of each sample was measured using a Synergy HTX Multi-Mode Reader (Biotek Instruments, USA) at an excitation/emission wavelength of 480 nm/520 nm.

### Preparation of dSIS/PLGA composite scaffold

In order to achieve satisfactory mechanical properties, the dSIS layer of the composite scaffold was formed by stacking four pieces of single-layer dSIS together (stacked and compressed in dehydrated status, and then lyophilized). PLGA (LA:GA = 85:15, ŋ = 3.1DL/g) was dissolved in 2,2,2-trifluoroethanol for 24 h to achieve a spinning solution with a concentration of 10% wt/v. The spinning solution was then electrospun onto the dSIS layer attached on a receiving roll, with a voltage of 15 kV, a flow rate of 2 ml/h, and a tip-to-collector distance of 20 cm. Electrospinning lasted for 2 h and resulted in a dSIS/PLGA composite scaffold. The prepared dSIS/PLGA scaffold was dried in vacuum at 35°C for 24 h to remove residual solvent and subsequently irradiated with 25 kGy for sterilization.

### Scanning electron microscopy

Morphology of dSIS/PLGA composite scaffold was characterized using scanning electron microscopy (SEM). Vacuum-dried samples were coated with 3 nm thick gold by a 108AUTO sputter coater (Cressington Scientific Instruments), and then observed with a S-520 microscope (HITACHI) at an accelerating voltage of 10 kV. The dSIS side and the PLGA side of the composite scaffold were both examined.

### Mechanical test

A universal testing machine (UTM6103, Shenzhen Suns Technology) was employed to characterize the mechanical properties of the composite scaffold. Test samples were cut into 10 mm × 60mm strips with eight replicas, and were hydrated in PBS for 10 mins before testing. The testing machine had a load cell of 200 N, and the tests were carried out with a clamp distance of 30 mm and a stretching rate of 0.6 mm/min. Tensile moduli, ultimate tensile strengths and failure strains were recorded.

### Cell culture

Primary rat esophageal smooth muscle cells (ESMCs) and esophageal epithelial cells (EECs) were purchased from Wuhan Procell Life Science Technology Co., Ltd. Cells from 2nd to 4th passages were seeded onto the composite scaffolds for co-culture. Scaffolds were cut into circular shapes that aligned with the growth areas of the culture plates, with either the dSIS side or the PLGA side exposed to the respective cells and culture media. ESMCs were seeded on the dSIS side of the scaffold and cultured in smooth muscle cell medium supplemented with 10% fetal bovine serum, 1% penicillin–streptomycin and 1% smooth muscle cell growth factor. EECs were seeded on the PLGA side of the scaffold and cultured in epithelial cell medium supplemented with 10% fetal bovine serum, 1% penicillin–streptomycin and 1% epithelial cell growth factor. Seeding density was 1 × 10^4^ cells/well for 96-well plates or 5 × 10^5^ cells/well for 6-well plates. Culture condition was maintained at 37°C with 5% CO_2_.

### Cell viability

Cells were co-cultured with scaffolds in 96-well plates for this analysis. Cells were also cultured in plain wells under the same condition to serve as controls. On Day 1, Day 3 and Day 7 of culture, assays were performed in selected wells with CCK-8 solution (10 µL for each well, and incubated for 2 h at 37°C). The optical absorbance of each well at 450 nm was measured by a spectrophotometer microplate reader (Biotek Instruments, USA).

### Immunostaining analysis

Cells were co-cultured with scaffolds in six-well plates for this analysis. Cells were also cultured in plain wells under the same condition to serve as controls. On Day 1, Day 3 and Day 7 of culture, cell-scaffold constructs were fixed with 4% paraformaldehyde for 30 min, washed three times with PBS for 15 min each, incubated in 1% Triton X-100 for 10 min, again washed three times with PBS, and blocked in 10% goat serum for 30 min. The serum was then drained and the samples were incubated overnight at 4°C with rabbit anti-actin antibody and mouse anti-CK-14 antibody for ESMCs and EECs, respectively. After three PBS washes, the samples were subsequently incubated in fluorescein isothiocyanate (FITC)-conjugated goat anti-rabbit IgG and FITC-conjugated goat anti-mouse IgG, respectively for 45 min at room temperature. Same washing was then performed and samples were immersed in DAPI solution for 5 min to stain nuclei. Fluorescence images were acquired with a TCS-SP8 laser scanning confocal microscope (Lecia, Germany), in which α-actin and CK-14 displayed green and nuclei displayed blue.

### Western blotting

Cells were co-cultured with scaffolds in six-well plates for this analysis with the above-mentioned methods. On Day 7 of culture, cell-scaffold constructs were subjected to RIPA lysis buffer to extract proteins. Total protein was quantified according to the instructions of BCA protein assay kit. 20 μg of proteins were then separated on 10% sodium dodecyl sulfate (SDS) polyacrylamide gel, and transferred onto nitrocellulose membranes. After blocking with 5% bovine serum albumin (BSA) for 1 h, the membranes were incubated with rabbit anti-actin antibody or mouse anti-CK-14 antibody overnight at 4°C. After five PBS washes, the membranes were then incubated with HRP-conjugated goat anti-rabbit IgG or HRP-conjugated goat anti-mouse IgG for 45 min at room temperature. Electrogenerated chemiluminescence (ECL) western blotting reagents were added to the membranes, and signals were then detected by an automatic chemiluminescence imaging system (Tanon 4600). The levels of detected proteins were assessed by measuring the integrated intensity of all the pixels in each band excluding the local background, normalized to the levels of GAPDH. Results were from at least three separate experiments.

### Statistical analysis

Data were reported as mean±SD, and statistical differences were analyzed by one-way analysis of variance (ANOVA). Differences were considered statistically significant at *P* < 0.05.

## Results

### Efficiency of SIS decellularization

**Figure 1.**
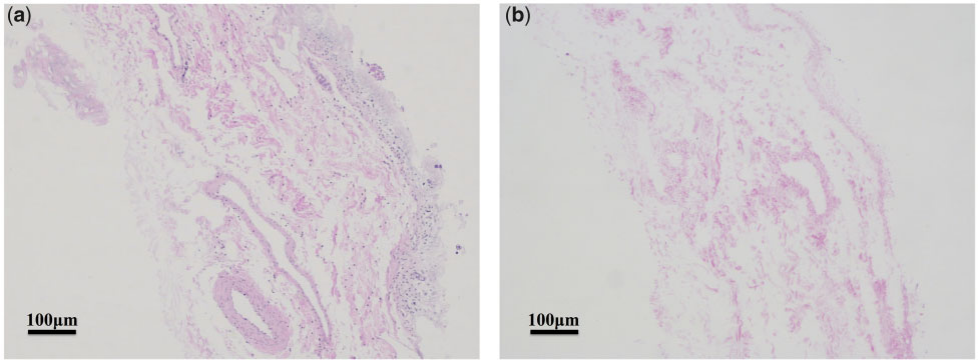
H&E staining images of (**a**) SIS and (**b**) dSIS

Figure 1 showed the histological images (stained with H&E) of the SIS tissues before and after the decellularization process. Only minimal amount of histologically visible nuclei could be seen in the dSIS, indicating satisfactory decellularization. At the same time, the extracellular matrix (ECM) appeared to have remained intact during the process. DNA quantification revealed that the DNA content of the SIS tissue was 835.7 ± 9.7 ng/mg before decellularization and 52.5 ± 1.2 ng/mg after decellularization, which meant more than 90% of the native porcine DNA was removed by the decellularization process.

### Physical characterization of dSIS/PLGA composite scaffold

**Figure 2.**
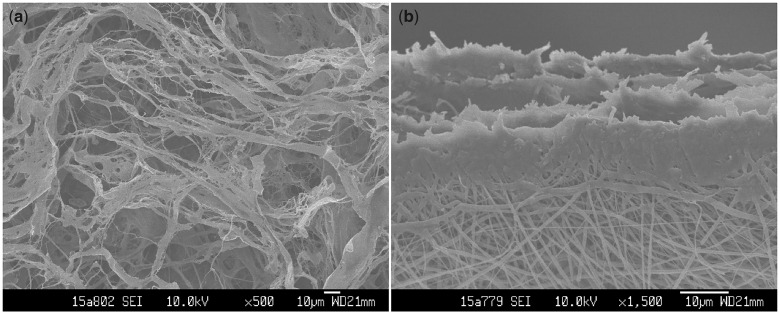
SEM micrographs of dSIS/PLGA composite scaffold: (**a**) principal view of the dSIS side; (**b**) the PLGA side with an angled view of the cross-section.

Morphologies of both sides of the dSIS/PLGA composite scaffold were shown in Fig. 2. It could be seen that the bi-layer scaffold was successfully fabricated, and the PLGA nanofiber layer attached firmly on the dSIS layer. The dSIS layer exhibited fibrous and porous structures (Fig. 2a), and the fact that it was formed by four pieces of single-layer dSIS was also confirmed (Fig. 2b). The PLGA nanofibers were shown to have diameters ranging approximately from 600 nm to 900 nm. The scaffolds showed typical elastomeric behaviors under tension. Table 1 listed the main mechanical properties of the scaffolds.

**Table 1.** Mechanical properties of dSIS/PLGA composite scaffold

Tensile modulus (MPa)	Ultimate tensile strength (MPa)	Failure strain (%)
21.1 ± 4.8	14.0 ± 2.9	26.9 ± 5.1

### Biochemical characterization of dSIS/PLGA composite scaffold

**Figure 3.**
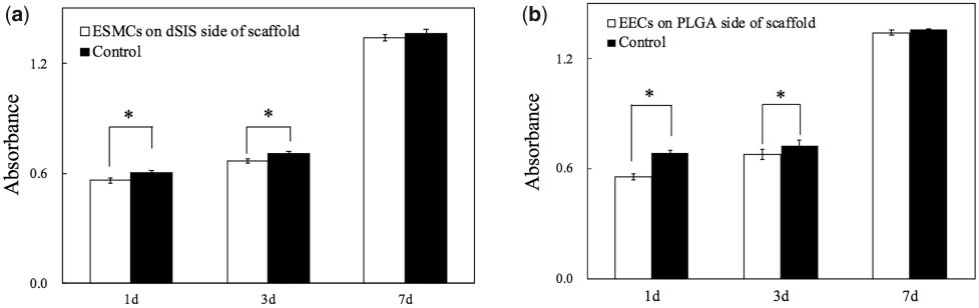
Cell proliferation measured by CCK-8 assays on Day 1, 3 and 7: (**a**) ESMCs on the dSIS side of scaffold; (**b**) EECs on the PLGA side of scaffold. *P < 0.05.

Figure 3 illustrated the absorbance values in the CCK-8 assays on different time points of culture. The results indicated evident cell proliferation of both ESMCs and EECs from Day 1 to Day 7 on dSIS and PLGA, respectively. There existed, however, an initial offset of cell numbers between the co-culture groups and the control groups, which later gradually diminished and became statistically insignificant by Day 7.

**Figure 4.**
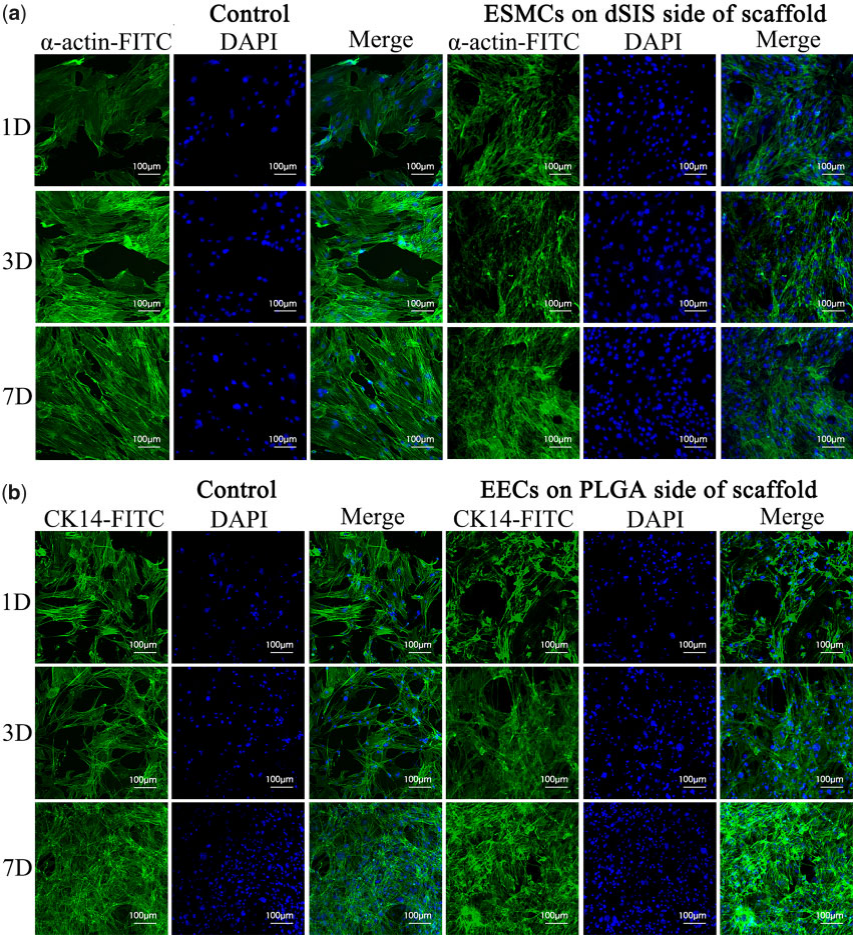
Immunofluorescent images of ESMCs and ECs on composite scaffolds: (**a**) ESMCs with anti-actin antibody on Day 1, 3 and 7. α-actin displayed green and nuclei displayed blue. (**b**) EECs with anti-CK-14 antibody on Day 1, 3 and 7. CK-14 displayed green and nuclei displayed blue.

Figure 4 displayed the immunofluorescent images of ESMCs and EECs on scaffolds on Days 1, 3 and 7. Images of respective control groups were also included for comparison. On the dSIS side of the scaffold, the ESMCs were found to have positively expressed α-actin (Fig. 4a), exhibiting their smooth muscle characteristics. On the PLGA side of the scaffold, CK-14 was also detected from the EECs (Fig. 4b), confirming their epithelial nature. The increased densities of nuclei in the fields of vision could again reflect the proliferation of both ESMCs and EECs.

**Figure 5.**
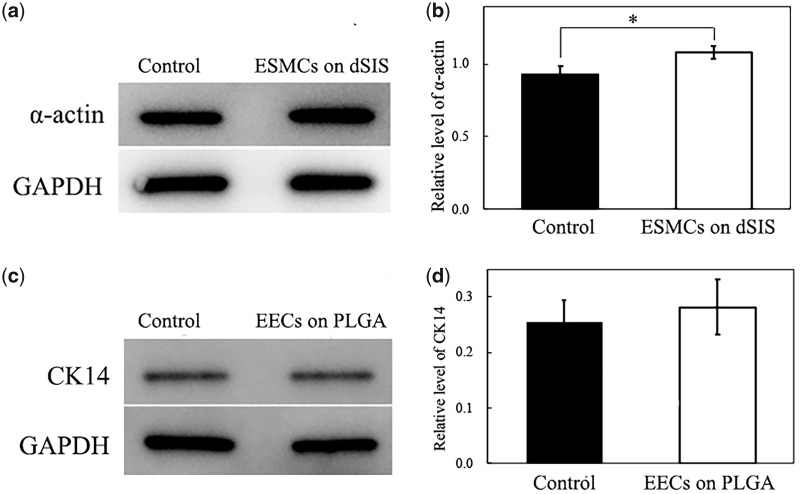
Western blotting analysis of ESMCs and ECs on the scaffolds: (**a**) Western blotting bands of α-actin and GAPDH of ESMCs on the dSIS side of scaffold and in control group. (**b**) Normalized intensities of α-actin bands. (**c**) Western blotting bands of CK-14 and GAPDH of EECs on the PLGA side of scaffold and in control group. (**d**) Normalized intensities of CK-14 bands. *P < 0.05.

Western blotting analysis (Fig. 5) was performed to quantitatively evaluate the cellular behaviors of the ESMCs and the EECs on the scaffolds. It could be seen that the ESMCs co-cultured on the dSIS were already secreting greater amount of α-actin on Day 7 than those in plain wells (Fig. 5a and b). Similar trend, i.e. EECs on PLGA secreting more CK-14 than the control group, was also observed, although not statistically significant (Fig. 5c and d).

## Discussion

Maximal decellularization is important for the dSIS to function as a template for tissue repair. Keane *et al.* [14] reported that a high level of decellularization and a low level of xenogeneic DNA in the biological scaffolds could promote expression of an M2 macrophage phenotype, which is critical to the scaffolds’ ability to promote constructive tissue remodeling when implanted in human bodies.

The decellularization process adopted in this study achieves 52.5 ± 1.2 ng/mg remnant DNA in the dSIS, which is satisfactorily in agreement with other researches [15, 16], suggesting suitability of the as-prepared dSIS for the intended application [17].

The design of the layered scaffold with dSIS and electrospun PLGA is relatively new. Syed *et al.* [18] reported an esophageal scaffold made of the same materials by similar techniques. In contrast to the scaffold in this paper, their esophageal scaffold placed dSIS at the intraluminal layer and PLGA at the extraluminal layer, and used PLGA mainly to mechanically reinforce the scaffold rather than promote epithelialization. The mechanical properties are indeed of great importance. The scaffold should not only be able to retain the sutures in the surgery, but also allow the repaired tissues to distend normally in the upper aerodigestive tract. The authors were also aware of the potentially insufficient mechanical properties of the dSIS. However, in order to form a mechanically stronger composite scaffold, the authors chose to stack and compress multiple sheets of pristine dSIS in the dSIS layer, instead of increasing the quantity (thickness) of PLGA in the PLGA layer. This approach is believed to have two advantages. Firstly, if the electrospun PLGA nanofibers were to reinforce the scaffold, usually they have to be well aligned and the reinforcement should be in a preferred orientation, whereas while using multiple sheets of dSIS the scaffold can enjoy an isotropic increase in the mechanical properties. Secondly, it has been reported that as the thickness of the electrospun layer increases, the residual charges accumulated on the already-deposited fibers would repulse the incoming fibers and hence make the later-deposited fibers less densely packed, jeopardizing the mechanical integrity of the scaffold [19, 20]. Therefore, the dSIS layer is considered more suitable as the mechanically dominant layer in this bi-layer design. The characteristics listed in Table 1 are comparable to those of Syed *et al.*’s [18] scaffolds in the reinforced orientation, and are also in line with those of the acellular dermal matrices (ADMs) [21] which have been clinically proven to be mechanically competent for hypopharynx and cervical esophagus reconstructions [22, 23], suggesting satisfactory mechanical properties of the composite scaffold.

ESMCs and EECs were respectively seeded on the dSIS layer and the PLGA layer, so that the biocompatibility of either layer could be independently assessed. The initial offset between the co-culture groups and the control groups in Fig. 4 was likely to be due to the seeding loss on the scaffolds. PLGA, as a synthetic material, has few natural recognition sites on the surface for the cells, and may suffer from a relatively low cell attachment ratio [24]. The number of EECs on PLGA on Day 1 seems to be approximately 20% lower than that in the control group. In contrast, the ECM proteins of dSIS could interact with the integrin receptors of cells [25, 26], which seem to have resulted in a better attachment ratio of ESMCs on dSIS. We are pleased to discover that both ESMCs and EECs have shown significant proliferation over the course of cell culture, indicating that the dSIS and PLGA materials are non-toxic and can well support cell growth.

The immunofluorescence and western blotting analysis not only double-confirms the increase in the cell numbers, but also reveals the phenotypic behaviors of ESMCs and EECs. α-actin is a major constituent of the contractile apparatus of the smooth muscle cells, and the successful expression of α-actin suggests the possibility of smooth muscle formation, and peristalsis where necessary. Meanwhile, CK-14 is a main protein to maintain the integrity and continuity of the epithelial tissues and positive expression of CK-14 indicates good phenotypic preservation of the EECs on the scaffold. The fact that by Day 7 the amount of α-actin and CK-14 in the co-culture groups is surpassing that in the control groups proves that either layer of the scaffold is favorable for its preferred cell type.

This study did not perform simultaneous co-culture of ESMCs and EECs on the same scaffold, which could be considered as a limitation. In the future, the authors will adopt a bioreactor to simultaneously seed and culture respective types of cells on their intended layers [27], so as to evaluate the whole scaffold under a close-to-*in vivo* condition. More importantly, should the scaffold alone not suffice in the clinical applications, cell-containing tissue-engineered grafts could also be developed by this means.

## Conclusion

In this study, a dSIS/PLGA composite scaffold is successfully fabricated. It is to the authors’ knowledge one of the first few scaffolds that possess layers intended for the mucosal and the muscular layers in hypopharyngeal and cervical esophageal tissues. It is mechanically comparable to the clinically approved ADM, but structurally more sophisticated. Preliminary results of seeding ESMCs and EECs on the respective layers have shown satisfactory cell–scaffold interactions, including cell proliferation and phenotypic behaviors. These results provide an important basis for the next-step *in vivo* studies, and bode well for the scaffold’s future clinical applications.
